# Investigation of High-Performance Electrode Materials: Processing and Storage Mechanism

**DOI:** 10.3390/ma15248987

**Published:** 2022-12-16

**Authors:** Qiang Chen

**Affiliations:** College of Material Science and Engineering, Zhejiang University of Technology, Hangzhou 310014, China; cq415@zjut.edu.cn

The scope of the Special Issue entitled “Investigation of High-Performance Electrode Materials: Processing and Storage Mechanism” includes the research on electrodes of high-performance electrochemical energy storage and conversion devices (metal ion batteries, non-metallic ion batteries, metal–air batteries, supercapacitors, photocatalysis, electrocatalysis, etc.), as well as the research on the structure of electrode materials and charge storage mechanism.

In recent decades, the development of electrochemical storage and conversion devices has been an important direction of clean and sustainable energy development [1,2]. High-performance electrode materials are the key to obtaining high energy density, high stability, and other indicators of devices, which has aroused great interest from scientists [3,4,5,6,7]. In particular, lithium cobalt oxide (1980), lithium iron phosphate (1997), and other high-performance electrodes discovered by John B. Goodenough, Stanley Whittingham, and Akira Yoshino (Nobel Prize in Chemistry, 2019) are used in lithium-ion batteries [8]. In fact, the structure/characteristics of electrode materials greatly affect the process of electrochemical storage and conversion [9]. For example, the charge transfer occurs in the redox process of electrodes, and the adjustable mechanism of the charge transfer process can be widely used in energy storage, electrocatalysis, etc. [10,11,12].

The key materials electrodes of clean energy technology and their device process still have many restrictive problems, such as poor stability, short life, low efficiency, limited operating conditions, and high cost [13,14]. This Special Issue highlights some of these methods to improve the electrochemical performance of electrode materials in different fields. Due to the difference of electrode structure/characteristics, the electrochemical storage of electrode materials is generally divided into battery-type, capacitive-type, and pseudocapacitive-type, which provides a basis for building different types of electrochemical energy storage devices [15]. Although lithium-ion batteries have achieved great success in achieving high energy density (~300 Wh kg^−1^), problems such as safety and lithium scarcity (20 ppm) seriously hinder their application [16,17]. Except to Li^+^, other metal ions (e.g., Na^+^ [18,19] K^+^ [20] Zn^2+^ [21,22,23] Mg^2+^ [24,25] Ca^2+^ [26] and Al^3+^ [27]) and non-metallic cations (e.g., H^+^ [28] and NH_4_^+^ [29,30]) that can charge the carrier battery/supercapacitor are an important supplement to traditional energy storage [31]. Host electrode materials need to match ions with different sizes/characteristics and face great challenges in terms of high performance and long-term stability.

To overcome these shortcomings, the widely used strategy relies on carefully selected electrodes and various forms of optimization. The host material with large pores is an important consideration in the selection of charge storage electrodes [32]. However, the charge transfer in the redox process is a challenge to the stability of the electrode structure [33]. In addition, surface engineering is necessary to improve the surface charge storage capacity of electrode materials and inhibit structural collapse, which can further expand its application possibilities [34]. The unclear charge storage mechanism is still an important factor limiting the actual development of devices. The development of in situ monitoring technology has opened a new window, which will gradually reveal the unknown charge storage mechanism [35]. Understanding the correlation between device performance and electrode characteristics, further developing the processing technology, and mastering the charge storage mechanism is a challenging and dynamic multidisciplinary field.

Traditional fossil energy is the basis of the development of modern human society, economy, and science and technology [36]. However, its reserves have been sharply reduced due to high development and consumption [37,38]. The development of high-performance electrodes involved in new energy storage and conversion equipment is urgent. The theme of this Special Issue is “Investigation of High-Performance Electrode Materials: Processing and Storage Mechanism”, which aims to collate and publish the work of high-performance electrode materials in the following energy storage fields: metal ion batteries, non-metal ion batteries, metal–air batteries, supercapacitors, electrocatalysis, etc. We would like to thank the authors for submitting their original research articles related to high-performance electrode materials. It is believed that these works will make important contributions to the improvement of electrode performance and the revelation of charge storage mechanism.
